# Magnetization transfer imaging using non‐balanced SSFP at ultra‐low field

**DOI:** 10.1002/mrm.30494

**Published:** 2025-03-17

**Authors:** Sharada Balaji, Neale Wiley, Adam Dvorak, Francesco Padormo, Rui P. A. G. Teixiera, Megan E. Poorman, Alex MacKay, Tobias Wood, Adam R. Cassidy, Anthony Traboulsee, David K. B. Li, Irene Vavasour, Steven C. R. Williams, Sean C. L. Deoni, Emil Ljungberg, Shannon H. Kolind

**Affiliations:** ^1^ Department of Physics and Astronomy University of British Columbia Vancouver British Columbia Canada; ^2^ Hyperfine Inc. Guilford Connecticut USA; ^3^ Department of Radiology University of British Columbia Vancouver British Columbia Canada; ^4^ Department of Neuroimaging King's College London London UK; ^5^ Department of Psychiatry and Psychology Mayo Clinic Rochester Minnesota USA; ^6^ Department of Pediatric and Adolescent Medicine Mayo Clinic Rochester Minnesota USA; ^7^ Medicine (Neurology) University of British Columbia Vancouver British Columbia Canada; ^8^ International Collaboration on Repair Discoveries University of British Columbia Vancouver British Columbia Canada; ^9^ Maternal, Newborn, and Child Nutrition & Health Bill & Melinda Gates Foundation Seattle Washington USA; ^10^ Department of Medical Radiation Physics Lund University Lund Sweden

**Keywords:** magnetization transfer ratio, multiple sclerosis, myelin imaging, portable point‐of‐care MRI, ultralow‐field MRI

## Abstract

**Purpose:**

Ultra‐low field MRI scanners have the potential to improve health care delivery, both through improved access in areas where there are few MRI scanners and allowing more frequent monitoring of disease progression and treatment response. This may be particularly true in white matter disorders, including leukodystrophies and multiple sclerosis, in which frequent myelin‐sensitive imaging, such as magnetization transfer (MT) imaging, might improve clinical care and patient outcomes.

**Methods:**

We implemented an on‐resonance approach to MT imaging on a commercial point‐of‐care 64 mT scanner using a non‐balanced steady‐state free precession sequence. Phantom and in vivo experiments were used to evaluate and optimize the sequence sensitivity and reproducibility, and to demonstrate in vivo performance and inter‐site reproducibility.

**Results:**

From phantom experiments, T_1_ and T_2_ effects were determined to have a negligible effect on the differential MT weighting. MT ratio (MTR) values in white matter were 23.1 ± 1.0% from 10 healthy volunteers, with an average reproducibility coefficient of variation of 1.04%. Normal‐appearing white matter MTR values in a multiple sclerosis participant (21.5 ± 6.2%) were lower, but with a similar spread of values, compared to an age‐matched healthy volunteer (23.3 ± 6.2%).

**Conclusion:**

An on‐resonance MT imaging approach was developed at 64 mT that can be performed in as little as 4 min. A semi‐quantitative myelin‐sensitive imaging biomarker at this field strength is available for assessing both myelination and demyelination.

## 1 INTRODUCTION

Modern clinical MRI is centered on the use of high field strength systems, usually 1.5 T or 3 T scanners, which are expensive to acquire and operate, and require extensive infrastructure including dedicated rooms, stable power, and access to cryogens. These factors have put these systems out of reach across many parts of the world. Ultra‐low field systems that operate at field strengths less than 100 mT^1^, ^2^, ^3^, ^4^, ^5^, ^6^ are more cost‐effective alternatives that are easier to operate, have lower infrastructural needs, and can be operated in diverse environments, including hospital intensive care units,^7^, ^8^ mobile vans,^9^ and resource‐constrained regions.^3^, ^10^ Lower safety concerns due to the lower field strength and smaller 5 Gauss line safety perimeter also allow these systems to be used in clinical applications where higher field MRI would otherwise be difficult, such as in non‐stable neonates^11^ or individuals with certain implants.^12^

The improved affordability and accessibility of ultra‐low field MRI systems has the potential to change health care delivery profoundly. Specifically, these systems can be deployed to areas that have traditionally had limited access to MRI, such as low and middle‐income countries,^13^ as well as rural, remote, and under‐serviced areas in higher‐income countries. Furthermore, the lower operating costs and increased access may also allow more frequent scanning of individuals with multiple sclerosis (MS), dementia, or other progressive neurological disorders. In research settings, frequent imaging may also be helpful in characterizing neurodevelopmental processes, such as myelination, which may be disrupted in neurodevelopmental disorders or affected by poor child health, malnutrition, or early disease and inflammation.^13^

Important to many of these applications is a reliable and quantitative myelin‐sensitive imaging biomarker. Brain development involves rapid changes in myelin content over the first few years of life^14^, ^15^, ^16^ and is influenced by a child's in utero and post‐natal environment, nutritional status, and other health and adversity factors. Demyelinating diseases such as MS can manifest with both focal and diffuse demyelination,^17^ which are related to clinical disability and cognitive performance.^18^, ^19^ Past approaches to imaging myelin have included quantitative and semi‐quantitative techniques like magnetization transfer (MT) imaging and multi‐component relaxometry^20^ as well as more qualitative approaches like ultrashort echo time and T_1_‐weighted imaging.

The magnetization transfer ratio (MTR) is an easily accessible imaging marker that is sensitive (although not specific) to myelin content.^21^ An MTR experiment involves the acquisition of two images, one with and one without MT weighting. Typically, MT weighting is achieved through the application of an off‐resonance prepulse to selectively saturate the macromolecular bound water pool. This is most commonly played out as part of a T_1_‐weighted spoiled gradient‐recalled echo (SPGR) sequence, which allows for time‐efficient, whole‐brain acquisitions. The non‐MT‐weighted image is acquired using the same readout sequence but without the off‐resonance pre‐pulse.^21^ This approach to MT weighting has recently been demonstrated at 55 mT.^22^ An alternative to this approach using rapid steady‐state free precession (SSFP) sequences can allow the generation of MT contrast with short acquisition times without the need for adding radiofrequency (RF) pre‐pulses. Although this approach is challenging at high magnetic fields due to specific absorption rate constraints, these concerns are absent at low and ultra‐low magnetic field strengths.

The presence of on‐resonance MT saturation in SSFP sequences was demonstrated by Bieri et al.,^23^ who introduced a method to create differential MT weighting using such sequences by modifying the amount of on‐resonance RF energy deposited. By using matched acquisitions with different amounts of RF energy deposition, the bound pool can be differentially saturated. In traditional MTR, the MT‐weighted image includes the effect of direct saturation, which is difficult to distinguish from the true MT saturation effect. An on‐resonance approach inherently accounts for direct saturation, so that the differential contrast is only due to differences in MT saturation.^24^ In practice, this was achieved by Bieri et al. using two balanced SSFP (bSSFP) acquisitions with different repetition times (TRs) and RF pulse widths.^24^ Differential MT weighting was explored in non‐balanced SSFP as well, with the strongest differential MT weighting found in bSSFP, followed by SSFP‐FID (FISP) and then SSFP‐Echo (PSIF).^25^ An approach for quantitative MT imaging using balanced and non‐balanced SSFP^26^, ^27^ has also been demonstrated, and such SSFP approaches have been illustrated in musculoskeletal,^28^ cardiovascular,^29^ and neurological^30^, ^31^ imaging applications.

Building on this high field work, we implemented an on‐resonance, non‐balanced SSFP approach to MT imaging at 64 mT by varying the amount of RF energy deposited between the two images. This approach was developed through phantom and in vivo experiments, and reproducibility was assessed in vivo in ten healthy volunteers, including two subjects scanned at different sites. Finally, the approach was also demonstrated in an example case of a person with MS.

## 2 METHODS

All experiments were performed on Hyperfine, Inc.'s 64 mT *Swoop®* system (hardware version 1.7, software version rc8.8 beta 1) using a head coil with one transmit and eight receive channels, unless stated otherwise. A PSIF sequence was created by modifying a FADE (fast double echo) sequence to capture only the echo, with the spoiling gradient in the readout direction, and used for all MT experiments. For in vivo scanning, all volunteers provided informed, written consent in accordance with the University of British Columbia's Clinical Research Ethics Board.

### 2.1 Phantom experiments

On‐resonance differential MT weighting using a PSIF sequence can be produced with two different approaches to change the RF energy deposited: by changing the RF excitation pulse flip angle (i.e., changing peak B_1_) while keeping the RF pulse width constant, or by changing the RF pulse width while keeping flip angle (peak B_1_) constant. These approaches were carried out experimentally in phantoms to determine the best approach to produce the highest differential MT weighting. The phantoms consisted of two vials of water (T_1_ = 4000 ms, T_2_ = 2000 ms at 1.5 T), two of dairy cream (whipping cream, 33% milk fat, T_1_ = 1147 ms, geometric mean T_2_ ˜71 ms at 1.5 T^32^) and two of hair conditioner^33^ (TRESemmé, Unilever PLC, London, UK, geometric mean T_1_ ˜600 ms, T_2_ = 158 ms at 1.5 T^34^). T_1_ relaxation times at 64 mT are expected to be shorter than at clinical field strengths,^35^ whereas T_2_ times have been found to be similar^36^ or slightly longer.^37^ The six phantom vials were bound together and placed in the head coil. Two experiments were carried out:

1. The flip angle of the RF pulse was varied from 1° to 420° in increments of 10°, and the RF pulse duration was set at 220 μs, resulting in 43 acquisitions. The flip‐angle acquisitions were interleaved as 1°, 420°, 10°, 410°, etc.
2. The duration of the RF pulse was varied from 40 to 220 μs in increments of 20 μs, and the RF pulse flip angle was set at 60° with a fixed TR, resulting in 11 acquisitions.

For both experiments, hard non‐selective RF pulses were used for 3D acquisitions, with the resolution set to 2 × 2 × 5 mm^3^ for axial acquisitions with field of view (FOV) = 180 × 220 × 200 mm^3^ (right–left [RL] × anterior–posterior [AP] × foot–head [FH]), TR = 12.03 ms, bandwidth = 18 kHz. Raw data from each acquisition were concatenated into a single dataset of dimensions 90 × 110 × 40 × 43 for Experiment 1 and 90 × 110 × 40 × 11 for Experiment 2, and reconstructed using the Berkeley Advanced Reconstruction Toolbox (BART).^38^ To estimate the contribution of each of the eight receiver coils to the signal, coil sensitivity maps were generated from flip angle (FA) = 60° images for Experiment 1, and from pulse width = 60 μs for Experiment 2, using BART's caldir^39^ function. The stacked data set from each experiment was then reconstructed using the pics command in BART with L1‐wavelet regularization across each image separately, and magnitude images were saved. L1‐wavelet regularization was chosen for its ability to denoise images in the compressed sensing reconstruction without blurring out details.^40^ For each experiment, regions of interest (ROIs) within each vial were manually drawn,^41^ and mean values from these regions were evaluated.

The dependence of the observed MT weighting on T_1_ and T_2_ relaxation times was also investigated using a quantitative phantom (CaliberMRI, Mini Hybrid model 137) consisting of 2 × 14 compartments with varying concentrations of MnCl_2_ and NiCl_2_, designed to cover a range of T_1_ and T_2_ values that span the range of healthy to diseased human tissue. Experiment 1 was repeated on this phantom, with the same scan parameters and reconstruction as described previously. This non‐MT phantom was not expected to show any apparent MT weighting, and if MT weighting was observed, it would be due to T_1_ and T_2_ effects. ROIs were manually drawn in the vials of known relaxation times, and mean values from these regions were evaluated at each flip angle.

### 2.2 In vivo experiment

Experiment 1 as described in Section 2.1 was repeated in one healthy volunteer to determine parameters for maximizing differential MT contrast in brain tissue. A T_2_‐weighted image was also acquired (3D fast spin echo sequence, 1.5 × 1.5 × 5 mm^3^, ˜6 min, TR/TE = 1600/170 ms, FOV = 180 × 220 × 200 mm^3^ (RL × AP × FH), bandwidth = 64 kHz) for anatomical segmentation and registration.

Raw data from the 43 flip angle acquisitions were reconstructed together as described in Section 2.1. The T_2_‐weighted image was brain‐extracted using HD‐BET,^42^ and a white matter mask was generated using Advanced Normalization Tools' (ANTs) *Atropos*.^43^ The T_2_‐weighted image was then registered to the FA = 60° image using a symmetric diffeomorphic transform,^44^ as the geometric distortion was pronounced due to the PSIF acquisition's low receiver bandwidth, and the white matter mask was warped to the FA = 60° image. A cerebrospinal fluid (CSF) mask was manually drawn on the FA = 60° image, and flip angle curves from within the white matter and CSF masks were determined.

### 2.3 In vivo reproducibility

The final parameters for in vivo scanning were MT_low_/MT_high_ = 60°/300°, RF pulse width = 220 μs, TR = 12.03 ms, resolution = 2 × 2 × 5 mm^3^, bandwidth = 18 kHz, FOV = 180 × 220 × 200 mm^3^ (RL × AP × FH), scan duration = 2 min for each of MT_low_/MT_high_ with a 10 s gap in between scans to allow for gradient cooling, for a total duration of 4 min.

To assess reproducibility, MRI data were acquired in 10 healthy volunteers (7 female/3 male, mean age 39 years, range = 25–76 years). T_2_‐weighted images (as described in Section 2.2) were acquired for anatomical registration and tissue segmentation, and MT_low_ and MT_high_ images were acquired with the parameters described previously. Subjects were then removed from the scanner and repositioned before re‐acquiring only the MT_low_ and MT_high_ images.

Raw MT data from each subject were reconstructed for each scan run using the same format as reconstructing the phantom data. Images of MT_low_ and MT_high_ were denoised using ANTs non‐local means (NLM) denoising.^45^ MTR maps were then calculated as MTR = (MT_low_ – MT_high_)/MT_low_ × 100%. T_2_‐weighted images were used to generate white matter masks and registered to each scan run's MT_low_ image as described in Section 2.2. Finally, overall white matter MTR values were extracted from each scan run for each subject and presented as (mean ± standard deviation) and as histograms. To further examine reproducibility in smaller structures, ROIs (genu, body, and splenium of the corpus callosum (CC), posterior limb of the internal capsule (PLIC), and corticospinal tract (CST)) were identified from the ICBM‐JHU atlas and warped to each subject's T_2_‐weighted image, and then to each run of the MT images. Mean values from each subject's ROIs were plotted in a Bland–Altman plot.

The scan–rescan coefficient of variation (CoV) for each subject's white matter and ROI‐specific MTR values was calculated as follows:

$$\begin{array}{cc} & \text{Scan}-\text{rescan}\;\mathrm{CoV}=\frac{\text{Standard deviation between scan and rescan}}{\text{Mean}\;\mathrm{MTR}\;\text{between scan and rescan}}\\ & \;\;\times 1.125\times 100\% \end{array}$$

The extra factor of 1.125 accounted for the small number of scans in the CoV calculation, which has the potential to bias CoV towards lower values.^46^, ^47^ The mean difference between each subject's scan and rescan white matter MTR values was also calculated.

To assess inter‐site reproducibility, one set of MT and T_2_‐weighted images was also acquired in 2 of the subjects in Gaborone, Botswana (Site 2, Hyperfine *Swoop®* hardware version 1.8, software version rc8.8 beta 1). The MT_low_ and MT_high_ images were reconstructed using the same format as described previously, and MTR values in white matter were calculated and presented here as (mean ± standard deviation) and as histograms compared to the values obtained in Vancouver, Canada (Site 1).

The offline reconstruction pipeline used for the MTR images did not include vendor‐specific corrections for gradient non‐linearities. To obtain undistorted images, we registered the MT component images to the T_2_‐weighted images using a symmetric diffeomorphic transform^44^ and Hamming‐windowed sinc interpolation. The reported MTR values were derived from original MTR images without any distortion correction, to not include smoothing effects from the interpolation step.

### 2.4 Demonstration in MS

MT imaging at 64 mT was also demonstrated in an example case of a person with MS. In one volunteer with relapsing–remitting MS (female, 48 years, expanded disability status scale score = 3.0) and one healthy volunteer (female, 52 years, from the reproducibility cohort), T_2_‐weighted (as described in Section 2.2) and T_2_‐FLAIR (1.7 × 1.7 × 5 mm^3^, TR/TE/TI = 3000/175/1130 ms, ˜8 min, FOV = 180 × 220 × 200 mm^3^ [RL × AP × FH]) images were acquired for lesion visualization, and MT imaging was performed with the sequence parameters described in Section 2.3.

Raw data from the MT images for each subject were then reconstructed using BART^38^ tools as described above in Section 2.3. Magnitude images of MT_low_ and MT_high_ were denoised with ANTs NLM denoising as before, and MTR maps were calculated. T_2_‐weighted images were brain‐extracted and used to generate white matter masks as in Section 2.2. T_2_‐weighted images were registered to the MT_low_ image, white matter masks were warped to MT_low_ image space, and MTR values in white matter were extracted without masking out lesions and presented as histograms and as (mean ± standard deviation). One lesion mask was manually drawn on the MS MT_low_ image, and its MTR was presented as (mean ± standard deviation).

## 3 RESULTS

**FIGURE 1:**
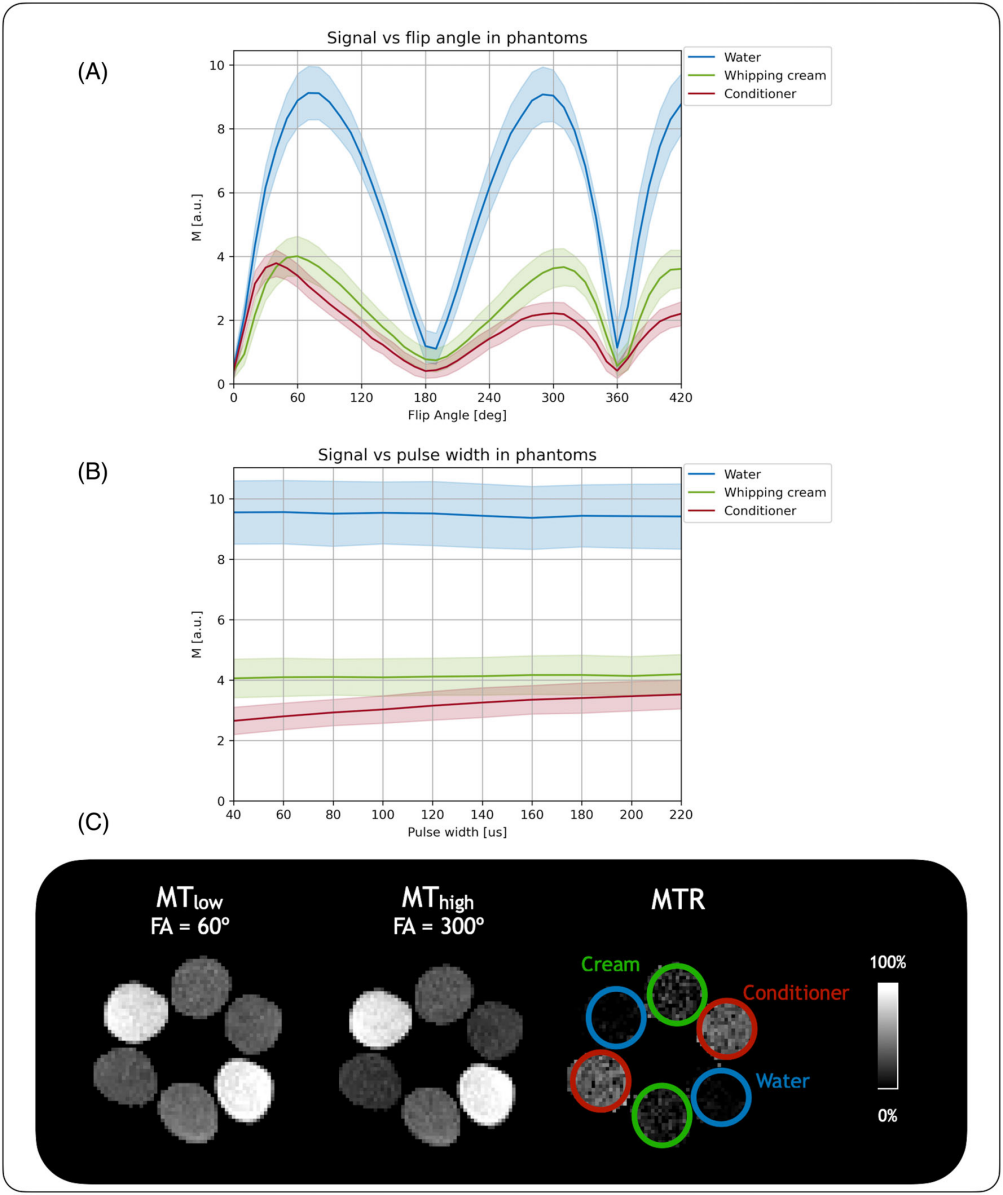
Phantom experiments in two vials of water, two of dairy cream, and two of hair conditioner to mimic different bound pool fractions. (A) Experiment 1 shows the effect of changing flip angle (FA) while maintaining radiofrequency (RF) pulse width and repetition time (TR). (B) Experiment 2 shows the effect of varying RF pulse widths while maintaining flip angle and TR. (C) shows a cross section of the vials at the two chosen flip angles and the resulting magnetization transfer ratio (MTR) map, with conditioner showing the highest MTR values, followed by cream, and then water with negligible values, as expected. [MT, magnetization transfer.]

Figure 1 shows the phantom experiments to demonstrate on‐resonance MT saturation using the PSIF sequence. The flip angle experiment curves were expected to be symmetric about 180° in phantoms with no bound pool, as the PSIF signal equation is symmetric about 180°,^48^ which was confirmed in the water samples, and asymmetry was expected to be introduced due to MT saturation in samples with a bound pool, which was confirmed in the dairy cream and hair conditioner samples. The flip angle experiment revealed a more pronounced differential MT effect at equidistant points about the symmetry point than in the pulse width experiment. Flip angles of 60° and 300° were chosen to provide the differential MT images as these points provided the same, maximal signal intensity in water, and were also close to the signal peaks for cream and hair conditioner. Figure 1C shows example images from using the two chosen flip angles and the resulting MTR map in phantoms.

**FIGURE 2:**
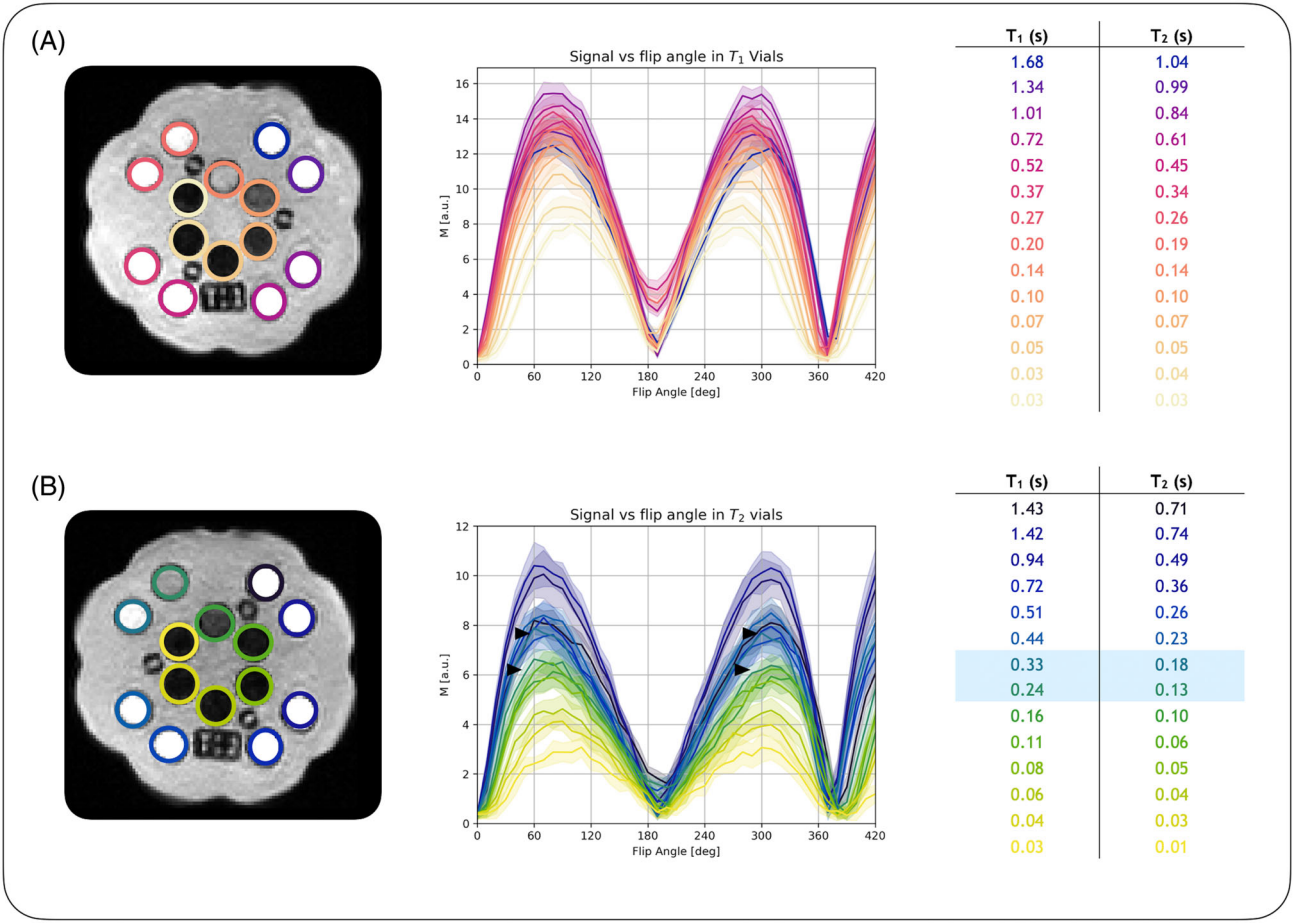
Two layers of the Caliber MRI mini‐hybrid phantom with (A) NiCl_2_ vials and (B) MnCl_2_ vials, along with tables of the T_1_ and T_2_ relaxation times of each vial at 64 mT for reference. The plots show the flip angle experiment conducted on this phantom, with each line representing one vial. The purpose of this experiment was to demonstrate that there is no apparent magnetization transfer (MT) effect in a non‐MT phantom, covering a large range of T_1_ and T_2_ relaxation times. It was expected that in this non‐MT phantom, the appearance of MT weighting would be caused by T_1_ or T_2_ effects. Note that there is generally little to no asymmetry around the symmetry point in the profiles for each vial, showing the lack of dependence on T_1_ and T_2_ effects on the observed MT weighting. The blue shaded rows indicate vials with relaxation characteristics closest to brain white matter (*black arrows*); these two vials show the expected symmetry at about 60° and 300°.

Results from repeating the flip angle experiment in the quantitative phantom (Figure 2) showed that although the location of the point of highest signal intensity varied between the vials due to differences in relaxation times, symmetry about the symmetry point was generally preserved, which suggests that T_1_ and T_2_ effects do not play a major role in the observed MT weighting. The MnCl_2_ vials most closely resembling white matter (marked on Figure 2) show the expected symmetry at 60° and 300°, indicating that these flip angles should provide the best differential MT weighting with highest signal intensity in white matter in vivo.

**FIGURE 3:**
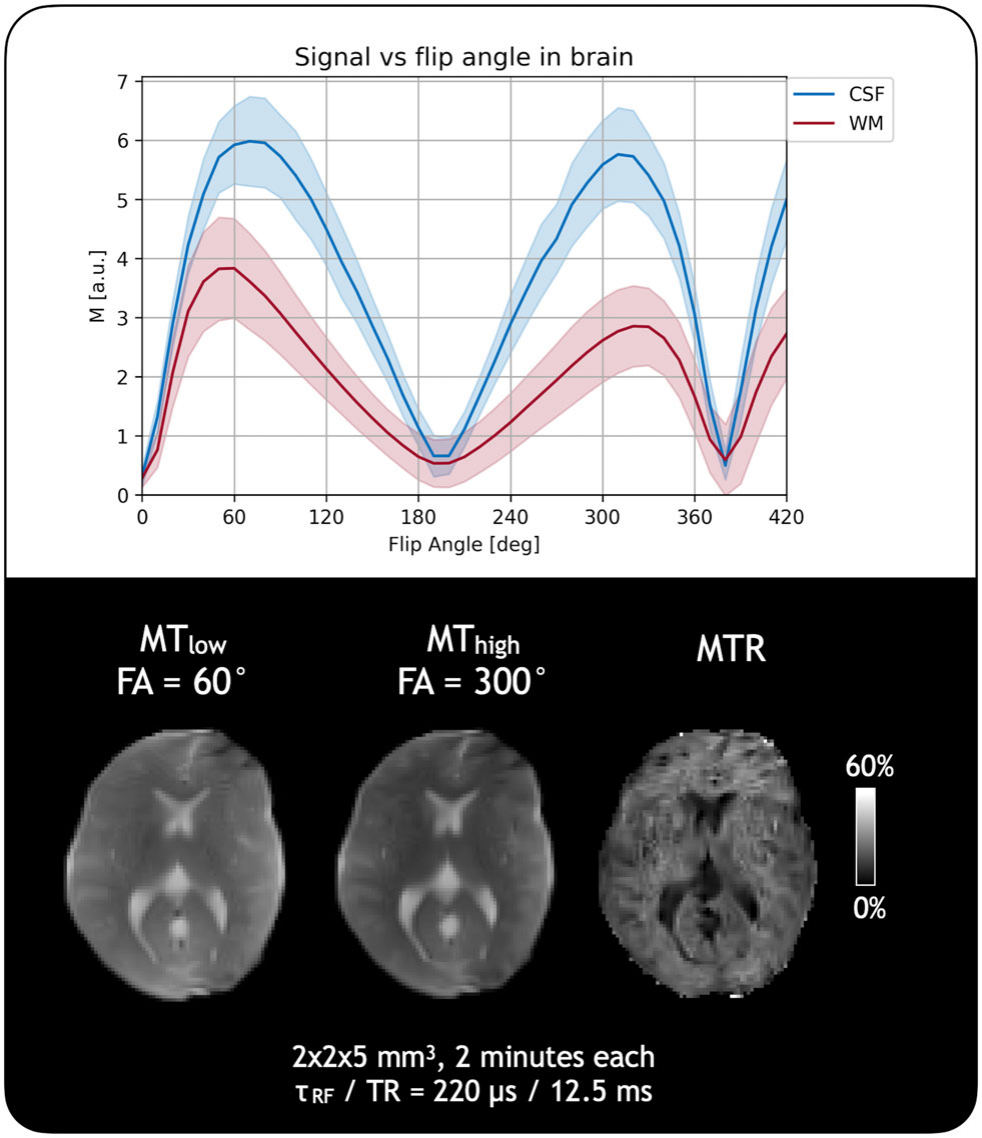
Magnetization transfer (MT) imaging in a healthy volunteer. (A) shows the full flip‐angle experiment run in a healthy volunteer, with flip angle curves shown for white matter (WM) and cerebrospinal fluid (CSF). Flip angle curves show the expected symmetry and asymmetry for CSF and white matter around the symmetry point. (B) shows a representative example of MT imaging in a healthy volunteer using in vivo parameters, along with the resulting MT ratio (MTR) map. Artifacts near the top right of the brain may be related to sensitivity maps, and the slightly patchy appearance of the MTR map may be related to post‐processing steps such as denoising.

Figure 3A shows the flip angle experiment carried out in a healthy volunteer, with curves presented for white matter and CSF. As expected, the flip angle curve in CSF resembled that of the water phantom, whereas white matter resembled that of the conditioner phantom. From these experiments, it was confirmed that flip angles of 60° and 300° would provide reasonable differential MT contrast, considering the possibility of flip angle inhomogeneity errors (about ±20° around 300°). Figure 3B shows a representative example of MT_low_ and MT_high_ images using flip angles of 60° and 300° and the resulting MTR map in a healthy volunteer. Artifacts near the top of the brain may be related to sensitivity maps used in our reconstruction, and the somewhat patchy appearance of MTR maps may be related to post‐processing steps such as denoising, which could be improved.

**FIGURE 4:**
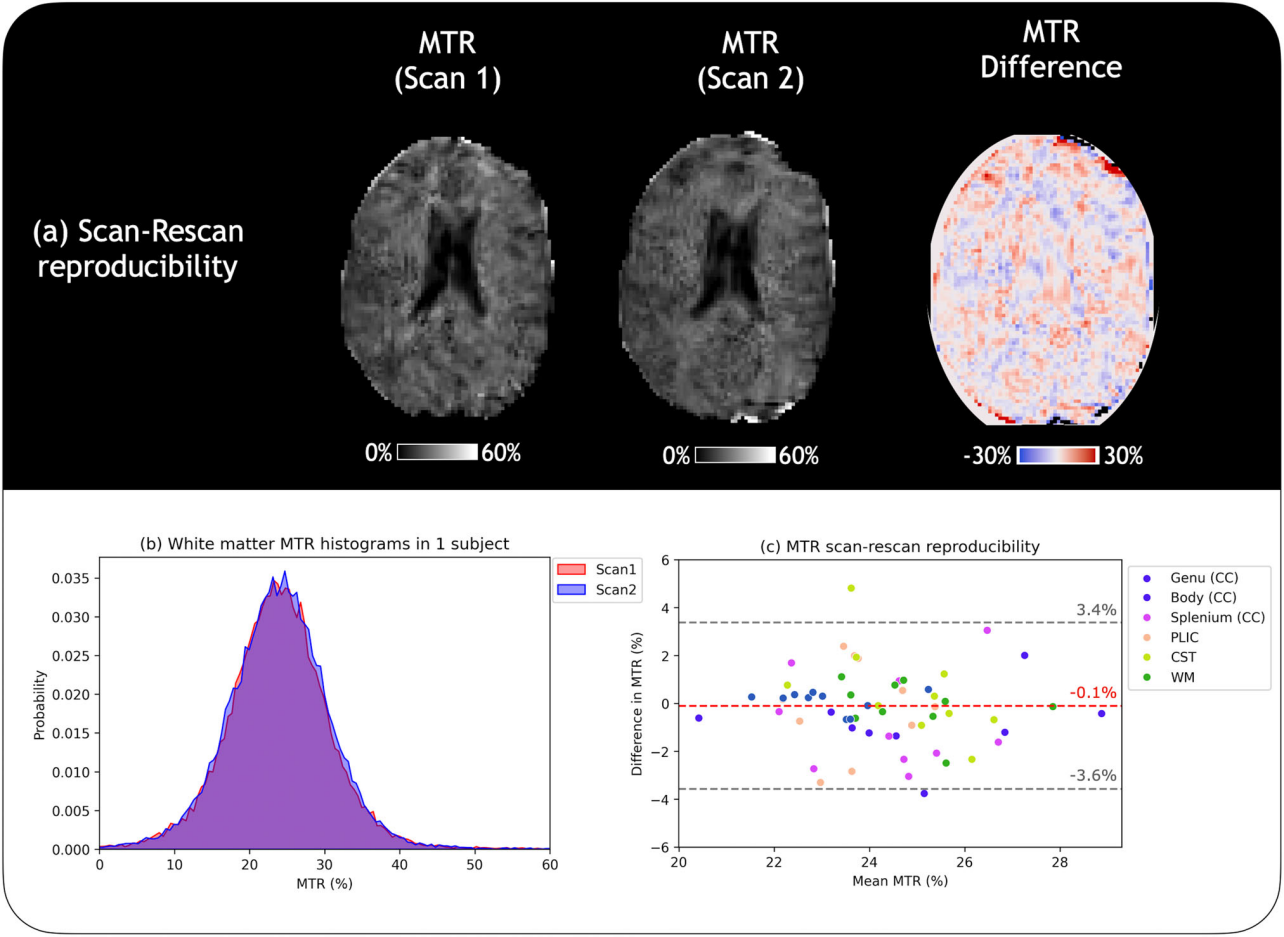
Reproducibility in healthy volunteers. (A) shows a representative subject's magnetization transfer ratio (MTR) maps from two scans with repositioning in between, and the difference map which shows no trend in spatial bias. The somewhat patchy appearance of the MTR maps may be related to post‐processing steps such as denoising. (B) is an example of white matter (WM) MTR values from the two scans, showing good overlap. (C) shows a Bland–Altman plot of mean scan and rescan MTR values from the genu, body, and splenium of the corpus callosum (CC), posterior limb of the internal capsule (PLIC), corticospinal tract (CST), and overall WM from the 10 healthy volunteers, showing reasonable reproducibility.

**FIGURE 5:**
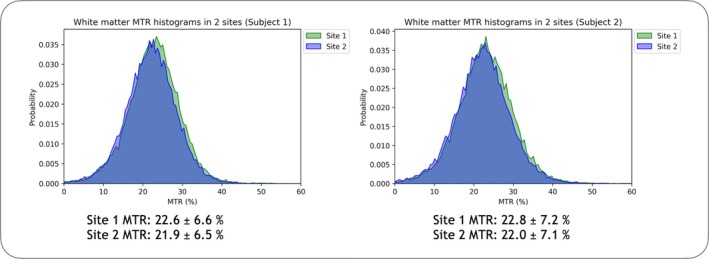
Histograms of magnetization transfer ratio (MTR) in white matter in 2 subjects from two different sites (Site 1 = Vancouver, Canada; Site 2 = Gaborone, Botswana), along with white matter means and standard deviations from each site. The histograms indicate good inter‐site agreement of MTR values.

Figure 4A shows a representative example of two MTR maps in one subject conducted with repositioning in between acquisitions, and the corresponding difference map of MTR values to demonstrate reproducibility. Although there are no clear trends of spatial bias in the difference map, voxel‐wise MTR differences were high as this implementation of MTR is relatively noisy, but MTR values were consistent when averaged over all white matter. Figure 4B shows an example histogram of white matter MTR in the same subject between the scan and rescan, showing good reproducibility. The Bland–Altman plot in Figure 4C shows the mean difference in MTR values between scan and rescan across the 10 healthy subjects over all the ROIs considered. MTR values in overall white matter across all subjects was 23.1 ± 1.0%, with an average scan–rescan CoV of 1.04% upon repositioning, and an average difference of 0.1% between the first and second scan. Average scan–rescan CoVs of the other ROIs considered, genu (CoV = 4.77%), body (CoV = 4.87%), splenium (CoV = 3.96%), posterior limb of the internal capsule (CoV = 3.44%) and corticospinal tract (CoV = 1.87%), also showed reasonable reproducibility. Figure 5 shows MTR histograms in white matter of 2 healthy volunteers scanned at two different sites, along with mean MTR values. The alignment of MTR histograms and mean values indicates adequate reproducibility between sites.

**FIGURE 6:**
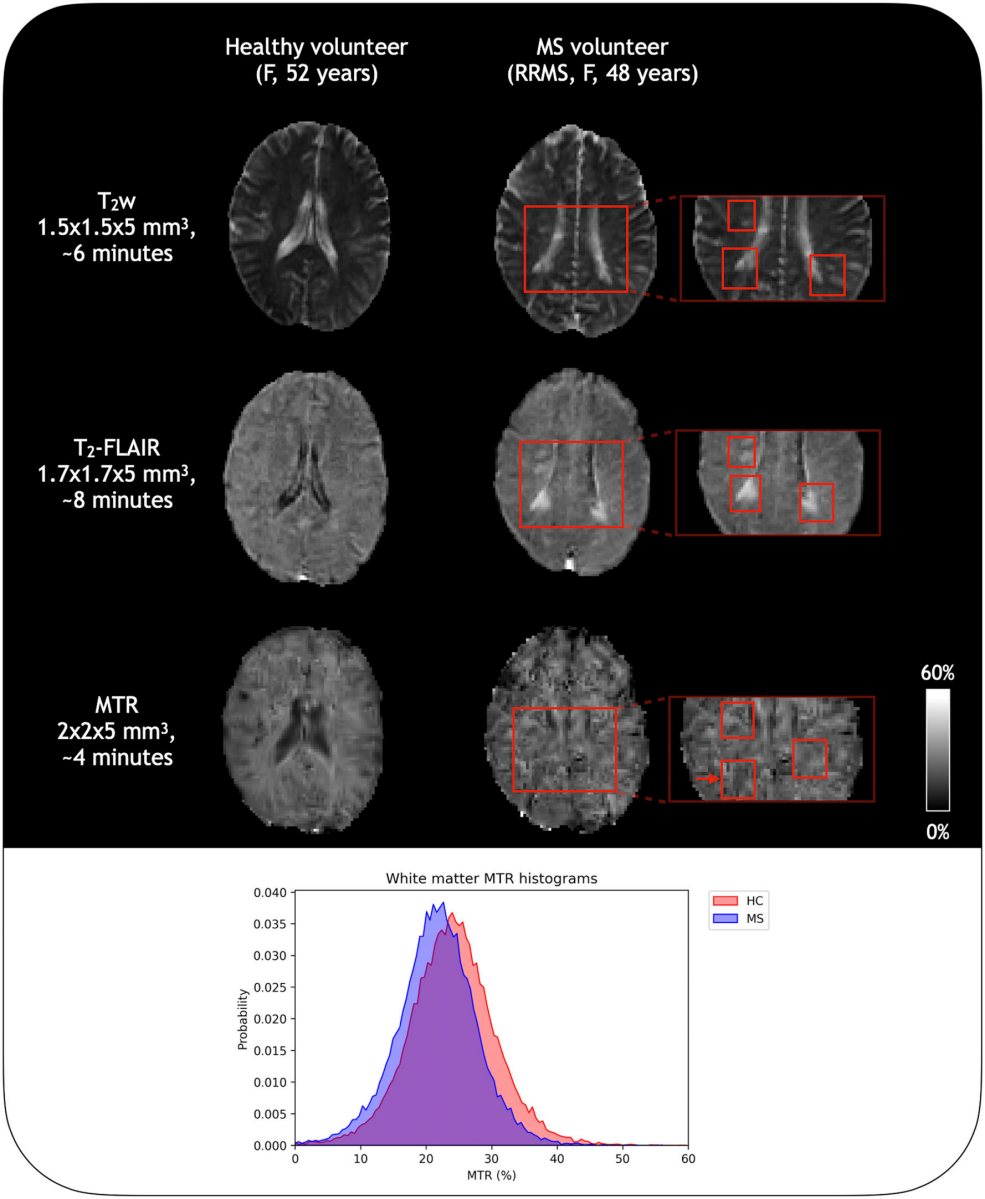
Magnetization transfer ratio (MTR) differences between a multiple sclerosis (MS) and age‐matched healthy volunteer. Red boxes on the images mark out visible lesions in the MS volunteer's T_2_‐weighted and T_2_‐FLAIR images that are also visible on the MTR map despite lower resolution. The MTR white matter histograms show lower MTR values overall in the MS volunteer than the healthy volunteer, which would be expected for diffuse demyelination. The lesion marked by the arrow showed a substantially lower MTR (16.9 ± 3.7%) than the normal‐appearing white matter (21.5 ± 6.2%). The somewhat patchy appearance of the MTR maps may be related to post‐processing steps. The MTR map of the healthy volunteer shows slightly higher values adjacent to the ventricles, likely due to a combination of ventricle pulsation, flow effects, and partial voluming with surrounding tissue. The MTR map of the MS participant shows cerebrospinal fluid values similar to white matter, likely due to partial voluming of the relatively narrow ventricles with the surrounding white matter. [FLAIR, fluid‐attenuated inversion recovery; T_2_w, T_2_‐weighted.]

Figure 6 shows images from an MS participant and an age‐matched healthy volunteer. The associated white matter histogram shows lower MTR values overall in the MS participant (MTR = 21.5 ± 6.2%) compared to the healthy volunteer (MTR = 23.3 ± 6.2%). The standard deviations indicate a similar spread in white matter MTR values in each person. The MS volunteer's white matter mask was not specifically designed to exclude lesions, but did avoid most. MTR for one lesion (marked by an arrow in Figure 6) was 16.8 ± 3.7%, substantially lower than the overall white matter.

## 4 DISCUSSION

In this work, we present an on‐resonance approach to MT weighting using a PSIF sequence at 64 mT through phantom experiments and demonstration in vivo in 4 min. The reproducibility of MTR values in healthy subjects was comparable to reproducibility at higher field strengths,^49^ and the relatively low variability of MTR values between healthy subjects was deemed sufficient to track changes to myelin content in neurodevelopment^14^ and neurodegeneration.^50^ The inter‐site reproducibility (Figure 5) was promising for tracking such changes even across different settings. The example case of a person with MS shows trends in line with other studies,^50^, ^51^, ^52^ where MTR values were lower in normal‐appearing white matter compared to healthy controls, with lesions showing focal areas of increased demyelination and particularly low MTR values. Diffuse changes in MTR in the normal‐appearing white matter are known to be related to clinical deficits in MS^52^; thus, even a relatively low‐resolution MTR measurement, as shown here, may have value for monitoring the progression of diffuse damage and could be made more sensitive to focal damage such as lesions with improved image signal‐to‐noise ratio (SNR). For tracking neurodevelopment in neonates, the recommended resolution is 3 × 3 × 5 mm^3^ to combat low SNR due to smaller head sizes and other environmental noise sources. It must be noted that this difference in resolution will affect the TR, which in turn affects the MTR values. MTR values are therefore not directly comparable at different resolutions; due to the sensitivity to changes in acquisition parameters,^24^ MTR is considered a semi‐quantitative biomarker for myelin content.

MTR values were lower than often reported at higher magnetic field strengths. MT imaging is highly dependent on T_1_ relaxation, as saturation is transferred only between the longitudinal components of the free and bound water pools.^21^ The MT ratio is governed by the rate of exchange between the bound and free water pools, as well as the longitudinal relaxation time of free pool protons, with shorter T_1_ times associated with lower MTR values.^21^ T_1_ relaxation is therefore an important consideration at low field, where T_1_ times in tissue are known to be shorter than at higher field strengths.^35^ Wang et al. showed that the observed change in T_1_ times of brain tissue as a function of field strength was due to changes in the T_1_ of the bound pool itself.^53^ At low field, a reduction in T_1_ of the bound pool allows for less time for magnetization exchange with the free water pool, resulting in generally reduced MTR values compared with high field under the same conditions.

MT contrast using on‐resonance SSFP has been shown in previous work to be more pronounced when using bSSFP sequences^25^ compared to PSIF, but with the point‐of‐care 64 mT system, heavy banding artifacts were found using bSSFP due to the high sensitivity of this sequence to B_0_ inhomogeneities. Although FISP would have been insensitive to B_0_ inhomogeneities and has been shown to have higher differential MT contrast and better gray/white matter contrast than PSIF,^25^ the FISP sequence had yet to be optimized for in vivo applications on the current system. The approach of varying the RF pulse width provided a less pronounced MT effect than varying the flip angle. Due to field inhomogeneities, the RF pulse bandwidth was required to be large, and RF pulse duty cycle and B_1_ amplitude limitations combined with the necessity of a short TR for steady‐state sequences meant that the RF pulse duration could not be made short or long enough at a fixed flip angle to provide a large differential MT weighting. Changing TR could also have been a possible approach to generate differential MT weighting. However, the insensitivity to TR changes that was exploited in Bieri et al.^24^ and Gloor et al.^26^ using bSSFP sequences does not extend to non‐balanced SSFP, where TR changes could lead to non‐MT signal variations. The PSIF sequence is particularly sensitive to TR changes due to changes in the T_2_‐weighted echo formation,^25^ so it was important to maintain a constant TR between the MT_low_ and MT_high_ acquisitions.

Motion and flow sensitivity are other important considerations that could impair image quality. Previous work with PSIF opted for 2D acquisitions to reduce flow‐related issues,^25^ but as our system allowed only 3D acquisitions at the time of these experiments, this was not feasible. Reduced sensitivity to flow could be achieved with flow compensation^54^ but at the expense of decreased SNR and increased TR. Due to the already low‐SNR nature of our PSIF acquisitions, flow compensation was not used in favor of higher SNR while maintaining a short scan duration. Motion such as CSF pulsation may also affect the image in the phase encode directions. Because of the lack of flow compensation, images (particularly regions around ventricles, such as periventricular lesions) must be considered carefully alongside qualitative images as in Figure 6, as the combination of flow/motion sensitivity and partial voluming may produce erroneous MTR values. Future work will investigate the use of motion correction methods for ultra‐low field applications to further improve image quality.^55^

Lower field strengths have advantages over high field, such as the lack of specific absorption rate constraints, making the current approach of varying the RF pulse flip angle as high as 420° feasible. However, due to the inherently lower SNR at low field, and particularly when using the PSIF sequence, the 300° flip angle images were often near the noise floor, resulting in some variability in image weighting due to difficulty in correcting environmental noise. Decreasing the receiver bandwidth and thereby increasing the SNR improved these reliability issues, although the relatively low bandwidth (18 kHz for voxel size = 2 × 2 × 5 mm^3^, FOV = 180 × 220 × 200 mm^3^) also resulted in stronger geometric distortions, which were corrected through image registration to the scanner‐reconstructed T_2_‐weighted image. The somewhat patchy quality of the MTR maps presented here likely stems from the NLM denoising in image space. Head position in the coil is an important consideration as well, as the 300° flip angle image is likely to descend closer to the noise floor with poor positioning, biasing MTR values. Some of the perceived MT effect may also have been due to B_0_ (and B_1_) inhomogeneity, resulting in imperfect on‐resonance RF pulses throughout the volume; this is noticeable in the flip angle experiments (Figures 1A, 2, and 3) in which the expected signal null at 180° was consistently offset to 190°, perhaps due to B_1_ errors. This may have also resulted in very slightly asymmetric peaks in both Figures 1A and 2 in non‐MT phantoms. Different approaches to image reconstruction may improve image quality. Figure S1 shows examples of other reconstruction approaches. Finally, although an off‐resonance approach as in Su et al.^22^ would have provided the most flexible solution with the ability to be added to any sequence, such as to increase apparent T_2_‐weighted contrast, given the restrictions of the system at the time of performing these experiments, modifying the RF energy deposition proved to be the more prudent approach.

## 5 CONCLUSION

This work introduces a novel approach for differential MT weighting and MTR imaging at 64 mT in as little as 4 min. In preliminary pilot data, we have demonstrated the method in healthy volunteers and an example case of a person with MS, showing reproducibility that bodes well for potential applications in characterizing neurodevelopment, neurodegeneration, and treatment response. This work contributes an important advancement in the state‐of‐the‐art of low field MRI, moving beyond conventional T_1_‐weighted and T_2_‐weighted imaging to offer a sensitive measure of myelin change.

## CONFLICT OF INTEREST

FP, RT, and MP have been employed by Hyperfine Inc.

## Supporting information


**FIGURE S1.** Comparison of the image reconstruction presented in the main paper, (A) (five iterations, L1 regularization factor = 0.02), with alternative reconstructions: (B) using no regularization resulted in an iterative sensitivity‐encoding (SENSE) reconstruction (five iterations), (C) using total variation (TV) regularization (20 iterations, TV regularization factor = 0.009), and (D,E) showing non‐uniform fast Fourier transform (nuFFT) followed by root sum of squares (RSS) of the individual coil images. In (A)–(D), non‐local means (NLM) denoising was performed after reconstruction. (E) shows nuFFT followed by RSS with no denoising for comparison. (A) is faithful to (B) and (D) with less noise, whereas (C) appears even more denoised and smoothed. Artifacts can be seen in (A)–(C) at the top right of the brain, related to the sensitivity maps. Improvements to sensitivity maps and improved reconstruction methods such as machine learning approaches may provide better image quality.
