# Supplementary material for: Magnetization transfer imaging using non‐balanced SSFP at ultra‐low field
Source: Magn Reson Med. 2025 Mar 17;94(2):602–14. doi: 10.1002/mrm.30494 (PMC12137778; doi:10.1002/mrm.30494)
Supplement: Supplementary file 1 — FIGURE S1. Comparison of the image reconstruction presented in the main paper, (A) (five iterations, L1 regularization factor = 0.02), with alternative reconstructions: (B) using no regularization resulted in an iterative sensitivity‐encoding (SENSE) reconstruction (five iterations), (C) using total variation (TV) regularization (20 iterations, TV regularization factor = 0.009), and (D,E) showing non‐uniform fast Fourier transform (nuFFT) followed by root sum of squares (RSS) of the individual coil images. In (A)–(D), non‐local means (NLM) denoising was performed after reconstruction. (E) shows nuFFT followed by RSS with no denoising for comparison. (A) is faithful to (B) and (D) with less noise, whereas (C) appears even more denoised and smoothed. Artifacts can be seen in (A)–(C) at the top right of the brain, related to the sensitivity maps. Improvements to sensitivity maps and improved reconstruction methods such as machine learning approaches may provide better image quality. [file MRM-94-602-s001.docx]

Supporting Information for

**Magnetization transfer imaging using non-balanced SSFP at ultra-low field**

Sharada Balaji, Neale Wiley, Adam Dvorak, Francesco Padormo, Rui P.A.G. Teixiera, Megan E. Poorman, Alex MacKay, Tobias Wood, Adam R. Cassidy, Anthony Traboulsee, David K.B. Li, Irene Vavasour, Steven C.R. Williams, Sean C.L. Deoni, Emil Ljungberg,
Shannon H. Kolind


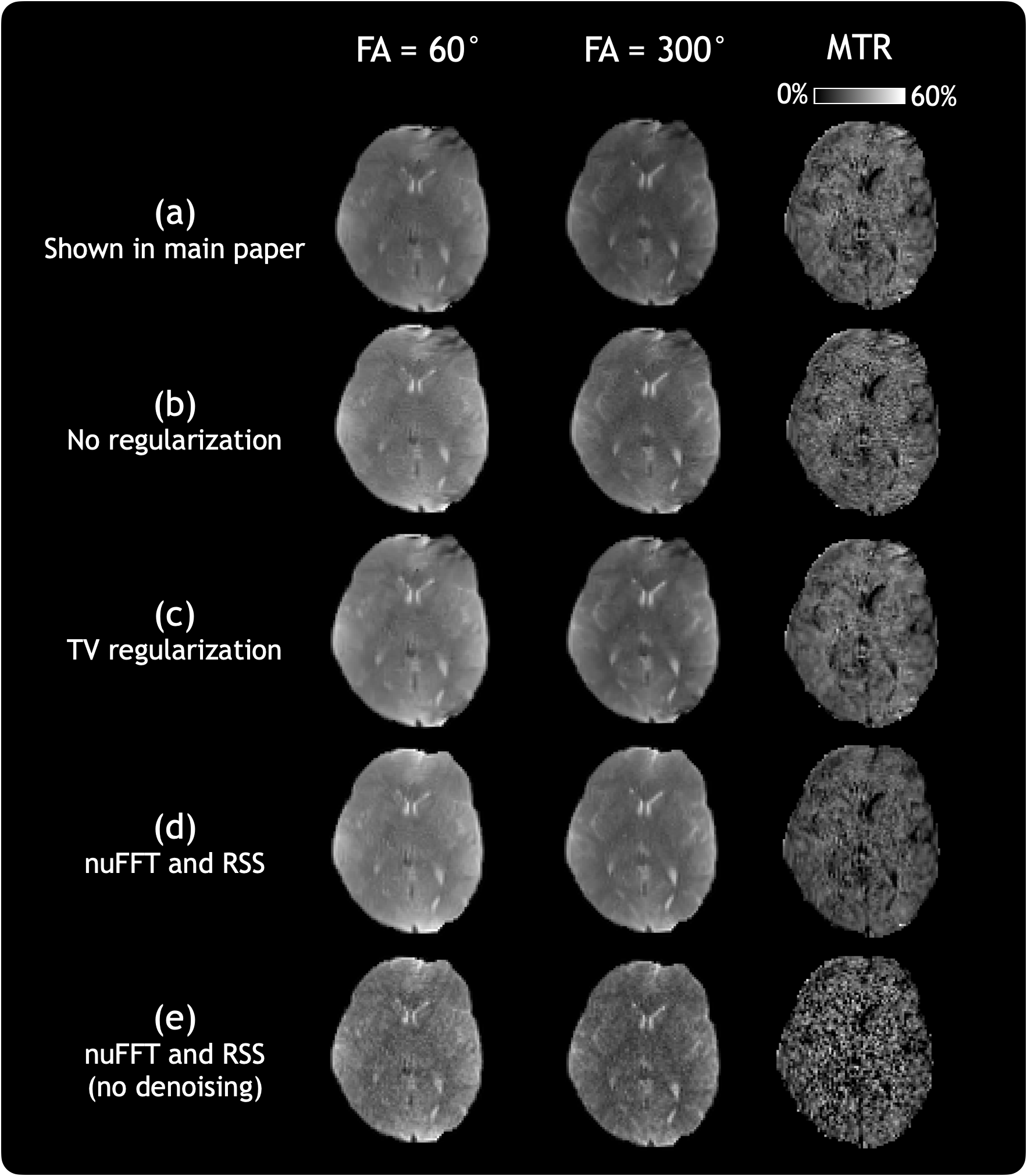


**Supporting Figure S1.** Comparison of the image reconstruction presented in the main paper (a, 5 iterations, L1 regularization factor = 0.02) with alternative reconstructions. (b) uses no regularization resulting in an iterative SENSE reconstruction (5 iterations), (c) uses TV regularization (20 iterations, TV regularization factor = 0.009), and (d, e) are non-uniform Fast Fourier Transform (nuFFT) followed by root sum of squares (RSS) of the individual coil images. In (a-d), non-local means (NLM) denoising was performed after reconstruction, and (e) shows nuFFT followed by RSS with no denoising for comparison. (a) is faithful to (b) and (d) with less noise, while (c) appears even more denoised and smoothed. Artifacts can be seen in (a-c) at the top right of the brain, related to the sensitivity maps. Improvements to sensitivity maps and improved reconstruction methods such as machine learning approaches may provide better image quality.
